# A review of portable quantitative and semi-quantitative devices for measurement of vitamin A in biological samples

**DOI:** 10.1016/j.crbiot.2022.04.003

**Published:** 2022

**Authors:** Samantha L. Huey, Jesse T. Krisher, David Morgan, Penjani Mkambula, Bryan M. Gannon, Mduduzi N.N. Mbuya, Saurabh Mehta

**Affiliations:** a Division of Nutritional Sciences, Cornell University, Ithaca, NY, United States; b Department of Large Scale Food Fortification, The Global Alliance for Improved Nutrition, Geneva, Switzerland; c The Global Alliance for Improved Nutrition, Washington, DC, United States; d Institute for Nutritional Sciences, Global Health, and Technology (INSiGHT), Cornell University, Ithaca, NY, United States

**Keywords:** Vitamin A, Retinol, Beta carotene, Portable devices, Field devices, Resource-limited settings

## Abstract

**Background:**

We catalog and summarize evidence of the analytical performance of portable quantitative and semi-quantitative devices for the assessment of vitamin A status and vitamin A deficiency (VAD) in various biological samples—including whole blood, plasma, serum, and milk—in addition to VAD determination by functional indicators such as pupillary response.

**Methods:**

We searched the literature for published research articles, patents, and information from manufacturers of mobile devices, particularly those appropriate for low-resource settings. The included devices were required to be portable (lightweight and ideally not needing a power outlet) and to measure vitamin A as well as define VAD. Eligible studies compared a portable device to a reference standard of high-performance liquid chromatography for blood and milk, or a Goldmann-Weekers dark adaptometer for eyes/vision. Where available, identified devices were compared with reference methods across several performance criteria. When possible, we compared the device’s performance reported in published studies against the stated performance criteria from the manufacturers’ websites.

**Results:**

We catalogued 25 portable devices for measuring vitamin A and/or VAD via biological samples. We also identified 18 comparison studies (plus associated reports) assessing nine methods: the iCheck Fluoro, iCheck Carotene, CRAFTi, Tidbit with or without the HYPER filtration system, custom field-friendly immunoassays, and microfluidic assays for blood; the iCheck Fluoro and iCheck Carotene for milk; and the Scotopic Sensitivity Tester-1 for eye function.

**Conclusions:**

The iCheck Fluoro and iCheck Carotene are commercially available for use and are acceptable for measuring vitamin A in blood and milk samples, according to the available validation data. Many of the other identified devices, including other portable fluorometers, photometers, immunoassays, microfluidics-based devices, and dark adaptometers, were proofs of concept and not yet commercially available. Furthermore, none of these other devices included manufacturer-described device performance criteria to compare with descriptions from experimental studies. Several gaps remain, including studies comparing the other portable devices against a reference standard, particularly for functional indicators of vitamin A status/deficiency; available manufacturer-reported device performance criteria against which to compare future results of investigations; and more comprehensive reporting of validation metrics including sensitivity, specificity, precision, and Bland-Altman analysis.

## Highlights

• Vitamin A deficiency is a major global health issue leading to poor health outcomes.
• Vitamin A assessment usually requires a centralized laboratory and equipment.
• Portable methods to assess vitamin A may overcome the limitations of laboratory-based testing.
• Blood, milk, and eye function may be used for vitamin A status measurement.

## Graphical abstract

Assessment of vitamin A concentrations in biological specimens or functional indicators of vitamin A status (severity of night blindness or lack thereof) require field-friendly methods for assessment to expand coverage, particularly in low-income settings. In this review, portable devices for measurement were compared with reference methods across key criteria. For example, portable devices to determine vitamin A concentrations in blood or milk samples were catalogued and compared with the reference method of high performance liquid chromatography, and a portable dark adaptometer to assess vision was compared against the reference of a Goldmann-Weekers dark adaptometer.

**Figure:**
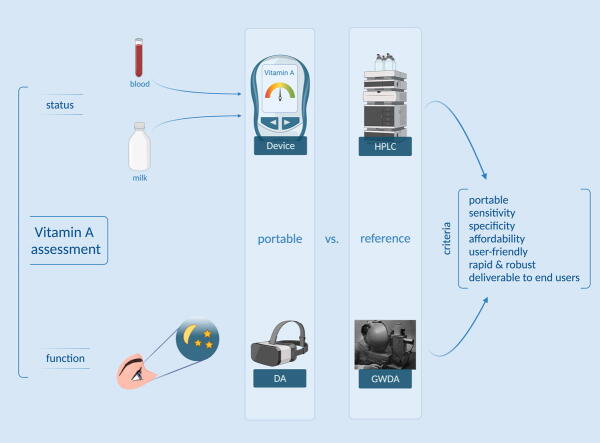

## Introduction/background

Vitamin A deficiency (VAD) continues to be a major global health issue leading to poor health outcomes, including night blindness, greater severity of measles infection, and higher mortality risk from infectious diseases (Tanumihardjo et al., 2016). Most existing analytical techniques to assess vitamin A status by measuring serum retinol or retinol binding protein require access to a sophisticated laboratory and equipment such as high performance liquid chromatography (HPLC) (de Pee and Dary, 2002). These methods require extensive sample preparation, are time-consuming, and are potentially prohibitively expensive, depending on the number of samples to be analyzed. Furthermore, VAD is more prevalent in lower income countries, where such laboratory resources may be limited or might not yet exist; in recent vitamin A surveys, <20% of pregnant women at risk have been covered by population surveys globally, possibly partly because of a lack of diagnostics (WHO, 2009).

Portable, field-friendly devices and tools for assessing vitamin A status in populations have the potential to overcome some of the limitations of traditional, laboratory-based testing. These methods may differ in their cost, accuracy, reliability, ease of use, and required consumables/reagents for performing the testing.

A review cataloguing the range of portable tests for vitamin A status and VAD in biological samples, and summarizing these devices’ performance with respect to a reference standard method, is not available. Therefore, the goal of this review was to enable current manufacturers to modify and improve their products according to the gaps identified herein, and to set design goals for new products meeting the current demands of industry, regulators, and other stakeholders.

## Materials and methods

In December 2020, we conducted a standardized search of the literature indexed in five databases (MEDLINE, EMBASE, World Health Organization Global Index Medicus, Scopus, and Web of Science) with no restrictions on language, location, or date of publication. We designed a search strategy for MEDLINE (PubMed) (**Supplementary Table 1**) and translated the search strategy for the remaining databases with guidance from the evidence synthesis specialists at Mann Library, Cornell University. We also used an online search engine to search for other sources such as manufacturers’ websites and patents, and we consulted with subject matter experts within our organizations to gain more information.

We catalogued any portable devices measuring vitamin A or vitamin A deficiency in biological samples, either as reported in studies or provided on manufacturers’ websites. We included both portable devices/methods measuring vitamin A status and devices/methods that indicated VAD. Initially, we considered devices measuring skin carotenoids, as shown in our search strategy; however, because of the lack of established guidance or consensus regarding the conversion of skin carotenoid measurements via Raman resonance spectroscopy (e.g., BioPhotonic Scanner (Pharmanex/Nuskin Enterprises, 2018)) to blood carotenoid measurements and overall vitamin A status (von Lintig, 2020), we determined that these devices were beyond the scope of the review.

The inclusion criteria for our analysis of device performance included certain study designs such as proof-of-concept development studies, method comparison studies, and diagnostic test accuracy studies; studies involving human participants (e.g., observational studies or randomized controlled trials) were considered if the authors described using a portable method for analyzing vitamin A in biological samples. Animal studies were also included. Eligible studies were required to measure vitamin A in any biological sample, including blood, eyes, or breast milk, with a portable device and to compare the device performance with that of a reference method, such as HPLC, depending on the sample type. Studies detailing field friendly methods of sample collection (e.g., dried blood spots) necessitating the use of a non-portable device or a laboratory for analysis were considered beyond the scope of this review.

We contacted the authors to request raw data or more information as needed. We also re-analyzed raw data, when available, as needed.

## Results and discussion

### Catalog of portable devices

**Fig. 1:**
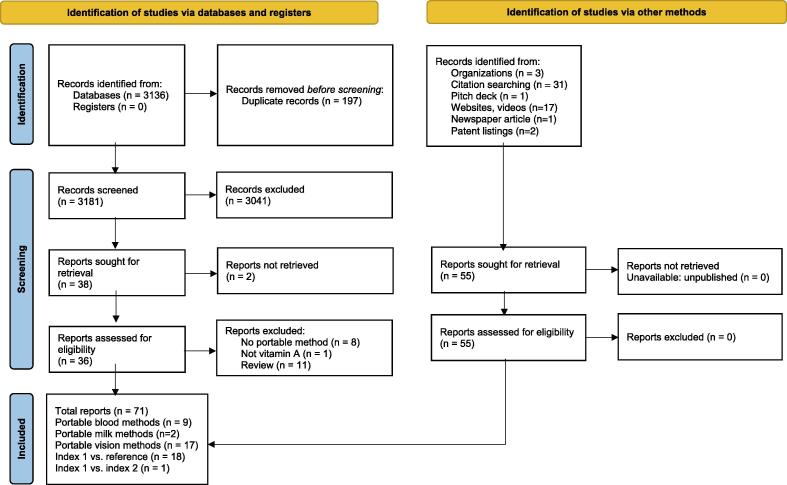
PRISMA Diagram for study identification and screening. ().

Adapted from Page et al., 2021

From our search (Fig. 1), we catalogued 25 portable devices, kits, and/or field-friendly assays able to assess a variety of biological sample and vitamin A biomarker types in Table 1a (blood, milk) **and** Table 1b (eyes/vision assessment).

**Table 1a:** Catalog of all portable devices quantifying vitamin A and vitamin A deficiency: blood, milk.

**Device (manufacturer)** **Principle/method** **# Tests per kit**	**Vitamin A biomarker**	**Pricing**	**Technical requirements** **Sample volume, preparation and setup** **Overall time required**	**Portability** **Included in kit** **Special storage conditions**	**Consumables:** **Reagents required** **Power source** **Shelf life**	**Operational range** **Quantification** **Outputs**	**Target setting** **Manufacturer support available** **Global availability**
iCheck Fluoro (BioAnalyt GmBH, Teltow, Germany) (BioAnalyt, 2021) Fluorescence 100 tests per kit	Retinol, retinyl palmitate, retinyl acetate and other esters	Pricing not published +	1 day training Whole blood, serum, breast milk: 0.5 mL; no preparation required <10 min +++	Compact and lightweight (11 × 4 × 20 cm); 0.45 kg Device + test kit (iEX MILA reaction vials), syringe None +++	Optional: 50 mL conical tubes, weighing dishes, reference samples Rechargeable battery 12 months at 20–30 °C, no direct sunlight, upright +++	50–3000 µg RE/L Quantitative Sample #, batch #, result, date, time (in transferred data); results (µg RE/L) are stored in the device and transferred to a PC via USB +++	Lab and field Yes >80 countries +++
iCheck Carotene (BioAnalyt GmBH, Teltow, Germany) (BioAnalyt, 2021) Photometry 100 tests per kit	Beta-carotene	Pricing not published +	1 day training Colostrum, cattle whole blood, cattle serum: 0.4 mL; no preparation required <10 min +++	Compact and lightweight (11 × 4 × 20 cm); 0.45 kg Device + test kit None +++	Optional: 50 mL conical tubes, weighing dishes, reference samples Rechargeable battery 12 months at 20–30 °C, no direct sunlight, upright +++	0.15–15 mg/L Quantitative Sample #, batch #, result, date, time (in transferred data); results (µg RE/L) are stored in the device and transferred to a PC via USB +++	Lab and field Yes >80 countries +++
CRAFTi (Eurofins CRAFT Technologies Inc., Wilson, NC, USA) (Chaimongkol et al., 2011, Eurofins Craft Technologies, 2020) Fluorescence NR	Retinol	Pricing not published +	Minimal training Serum: 25 µL; requires serum separation 30 min ++	Compact and lightweight (13 × 16.5 × 35 cm); 2.1 kg NR NR ++	Fluorometer cuvettes or Durham tubes Battery (12 V and inverter) or line current (115–230 V) Fluorescent dye fades; must check periodically ++	0.5–1.5 µmol/L Quantitative Fluorescence readings; no detail on data appearance ++	Lab and field Yes NR ++
Tidbit (Lu and Erickson, 2017, Lu et al., 2017), ± HYPER filtration system (Lu et al., 2018) (Cornell University, Ithaca, NY, USA) Fluorescence, multicolor lateral flow NR	RBP	Estimated: $95 manufacturing cost; $1.50 per test; Using HYPER platform, <$1 per test ++	Meant for consumer, clinical, and research use Serum: 15–20 µL, separated from RBCs with a centrifuge Whole blood: 60 µL, using HYPER (Lu et al., 2018) Serum: 15 min Whole blood: 5–20 min for HYPER separation) +++	NR; meant for field use Tidbit reader, disposable test strip(s) None +++	Lightening-Link Conjugation Kits (Innova Bioscience Ltd., HF180 cards (EMD Millipore); Running buffer (60 µL) Battery; connect to mobile device, Wi-Fi optional NR +++	2.2–20 µg/mL (0.10–0.95 µmol/L) Quantitative Result from each sample to smartphone (Nutriphone app) or laptop; stores results internally via 16-GB SD card +++	Lab and field Corresponding authors: David Erickson or Saurabh Mehta; not commercially available NR ++
Electronics-enabled (EE)-µPAD (Diagnostics for All) (Lee et al., 2016) Paper-based microfluidics for immune detection NR	RBP	Estimated: $20 for prototyping; $0.41 per test, but price expected to decrease below $10 per unit and at $1 per biomarker per unit ++	Meant for clinicians and researchers Whole blood: 35 µL; no preparation required 13 min +++	Size of a credit card NR NR ++	µPAD Battery Stored at room temperature in desiccator box until use +++	∼10 µg/mL to < 70 µg/mL, according to on graph (Fig. 5) Quantitative Measurements are wirelessly transferred to a mobile phone application that geo-tags the data and transmits it to a remote server for real time tracking of micronutrient deficiencies; NFC-enabled smartphone required +++	Resource limited settings Yes, but not commercially available NR ++
RBP-EIA (Scimedx Corp., Dover, NJ, USA) (Hix et al., 2004, Hix et al., 2006, 2020) Antigen competition assay 8–96 tests per kit	RBP	Estimated: <$3.00 per test; pricing not published ++	Meant for health care workers Serum: 10 µL; portable battery-operated centrifuge to separate whole blood, vortex serum samples and store on ice until assay completed 40 min ++	Size of 96-well plate; requires sink for washing step NR NR ++	Well plate, monoclonal anti-RBP antibody, wash buffer, substrate None required NR ++	10–40 µg/mL (0.48–1.92 µmol/L) Quantitative Read optical densities using EIA plate reader (Revelation, Dynex) ++	Lab and field Yes NR +++
Antigen-antibody reaction based on liquid-semisolid phase (custom) (Ciaiolo et al., 2015) Custom antigen–antibody reaction based on liquid-semisolid phase + visualization system NR	RBP	Pricing not published +	Meant for research or diagnostics Serum: 5 µL; requires serum separation 30 min ++	Petri dishes, pipette, portable viewer, and glass gel holder NR NR ++	Dilution: PBS due to high protein concentration; gel; antibodies None required NR ++	Depends on time for reaction; range 64 mg/L to 1 mg/L Qualitative, semi-quantitative Immunoprecipitates, scored as: “-, +, ++, +++, ++++” ++	Lab and field Corresponding author: Carlo Ciaiolo; but not commercially available NR ++
RID plate reader (The Binding Site, San Diego, CA, USA) (Hix et al., 2004, The Binding Site, 2020) Radial immunodiffusion of antigen–antibody precipitin rings 1–3 plates per kit	RBP	Pricing not published +	Meant for researchers Serum: 5 µL; requires serum separation Incubation for 3 days +	Compact and lightweight (22 × 14 × 16 cm, 1.14 kg) User guide and installation CD, USB-A to USB-B cable, power supply, plate reader calibration plate Indoor use only, altitude < 2000 m, 5–40 °C, relative humidity ≤ 80% at < 31 °C (or ≤ 50% if > 31 °C) ++	Microsoft Windows computer Power adapter, USB port NR (warranty: 1 year) ++	Depends on analyte; range for RBP not reported Quantitative Precipitin ring diameters, mm ++	Lab Yes NR ++
Reference method: HPLC Chromatography		$20000–$50000 per machine $50–$100 per test +	Meant for researchers, ≥500 µL, requires HPLC solvent and other preparation ≥65 min +	Not portable n/a Controlled conditions +	Can be used for different analyses or when the procurement of vials is difficult Requires external power source Requires routine maintenance +	Depends on analyte Quantitative Exact concentration output on attached computer, chromatogram with quantified absorbance for vitamin A concentration +++	Lab Yes +++

**Notes:** EIA, enzyme immunoassay; HPLC, high-performance liquid chromatography; NR, not reported; RBP, retinol-binding protein; RID, radial immunodiffusion.

+++ = best.

++ = acceptable.

+ = not acceptable.

**Table 1b:** Catalog of all portable devices quantifying vitamin A and vitamin A deficiency: eyes.

**Device (manufacturer)** **Principle/method**	**Vitamin A biomarker**	**Pricing (estimated, list price from manufacturer website)**	**Technical requirements** **Sample site; time for full charge**	**Portability** **Included in kit**	**Power source** **Usage duration per charge**	**Slit lamps:** **Magnification** **Dioptic range** **Interpupillary range** **Slit image width(s)** **Filters**	**Target setting** **Manufacturer support available** **Global availability**
BA 904, BA 904C (Haag-Streit, Harlow, Essex, UK) Haag-Streit, 1900 Slit lamp	Ocular morbidities	Pricing not published +	Meant for researchers and ophthalmologists Anterior segment; 4–5 h ++	“Lightweight” BA 904: head and chin rest stand, two energy packs, charger, power supply and large case; BA 904C: two energy packs, charger, power supply, parking unit and small case +++	Batteries and chargers 45 min +++	10×, 16× −8 to + 8 53–95 mm NR Blue, yellow +++	Field, clinic Yes Yes +++
Hand-held digital slit lamp (HSL-100, HSL-150) Portable slit lamp (Heine®) (Melo et al., 2004, Heine Optotechnik GmbH, 2018) Slit lamp	Ocular morbidities	Pricing not published +	Meant for researchers and ophthalmologists Anterior segment; NR ++	70 g BETA4 SLIM NT rechargeable handle and NT4 table charger (included reducer insert), spare bulb, hard case +++	Rechargeable or battery handle NR +++	10×, 16× NR NR 10 × 0.2 to 14 × 4 mm Cobalt blue +++	Field, clinic Yes Yes +++
Portable slit lamp (SL-17) (Kowa Ophthalmic Diagnostic Products, Torrance, CA, USA) (KOWA New Lighter) Slit lamp	Ocular morbidities	Pricing not published +	Meant for researchers and ophthalmologists Anterior segment; NR ++	<800 g; 220 × 95 × 220 mm 4 AAA batteries, dust cover, stand, instruction manual; optional: forehead rest, camera connection adapter +++	4 AAA rechargeable or dry cell batteries 130–140 min +++	10×, 16× NR 50–72 mm 1 × 1, 0.15, 0.5, 0.8, 1.6, 12 Cobalt blue +++	Field, clinic Yes Yes +++
Binocular hand held biomicroscope slit lamp (PSL One, PSL Classic) (Keeler, Malvern, PA USA) Slit lamp	Ocular morbidities	Pricing not published +	Meant for researchers and ophthalmologists Anterior segment; 2.5 h +++	900 g; 238 × 116 × 210 mm Base charger unit, power supply, user instructions, lens cloth +++	AC-powered 50 min ++	10×, 16× −7 to + 7 50–72 mm 0.15 mm, 0.5 mm, 0.8 mm and 1.6 mm slits, 12 mm circle and a 1 mm square Red free, blue, neutral density 0.8 and clear +++	Field, clinic Yes Yes +++
Handheld Slit Lamp S200 (Digital Eye Center, Miami, FL, USA) (Digital Eye Center, 2021, Digital Eye Center, 2021, Digital Eye Center, 2021) Slit lamp	Ocular morbidities	$2090 ++	Meant for researchers and ophthalmologists Anterior segment; NR +++	40 g; NR Universal smartphone adapter, metallic case, accessories. +++	Rechargeable battery 7 h ++	10×, 16× Diopter adjustment (not specified) 50–74 mm Slit width adjustment (not specified) Red free, green, cobalt blue, heat absorption, clear, neutral density +++	Field, clinic Yes Yes +++
Handheld Slit Lamp S2 (Digital Eye Center, Miami, FL, USA) (Digital Eye Center, 2021, Digital Eye Center, 2021, Digital Eye Center, 2021) Slit lamp	Ocular morbidities	$1500 ++	Meant for researchers and ophthalmologists Anterior segment; NR +++	750 g; 19 × 105 × 230 mm Smartphone adapter, metallic case, accessories +++	Rechargeable battery 2 h ++	10×, 16× −5 to + 5 45–70 mm 0–10 mm Heat-absorption, gray, red-free, cobalt blue +++	Field, clinic Yes Yes +++
Digital portable slit lamp Microclear Hyperion (Digital Eye Center, Miami, FL, USA) (Digital Eye Center, 2021, Digital Eye Center, 2021, Digital Eye Center, 2021) Slit lamp	Ocular morbidities	$3800 ++	Meant for researchers and ophthalmologists Anterior segment; NR +++	600 g; NR 4″ touch screen, 16 GB internal memory, two lithium batteries (4 h each), software and manual +++	Rechargeable battery 4 h +++	10× NR NR 0–10 mm Heat-absorption, gray, red-free, cobalt blue +++	Field, clinic Yes Yes +++
Hand held slit lamp (SL280) (Opticlar, Poole, Dorset) (Optical Visionmed) Slit lamp	Ocular morbidities	$3900 ++	Meant for researchers and ophthalmologists Anterior segment; 2 h +++	880 g; 163 × 124 × 205 mm Base plate, aluminum case +++	Rechargeable battery 6 h +++	10×, 16× −7 to + 7 50–78 mm 0.15/0.5/0.8/1.6 mm. Circle 12 mm dia. 1 mm square Green (red free), cobalt blue, neutral density 0.8, clear +++	Field, clinic Yes Yes +++
Portable slit lamp (PSL) (Reichert Technologies Inc., Depew, NY, USA) (Reichert Technologies, 2021) Slit lamp	Ocular morbidities	Pricing not published +	Meant for researchers and ophthalmologists NR +	680 g; fits in palm of hand Two batteries, battery charger +++	Rechargeable batteries 2 h +++	10×, 16× −7 to + 7 50–70 mm 0–11 mm Cobalt blue, red free, color temperature conversion +++	Field, clinic Yes Yes +++
Handy Slit Lamp XL-1 (Shin-Nippon by Rexxam Co., Ltd.) (Rexxam, 2021) Slit lamp	Ocular morbidities	Pricing not published +	Meant for researchers and ophthalmologists Anterior segment; NR +++	700 g (195 × 105 × 230 mm) Carrying case, one battery, battery charger, forehead support, diopter adjustment bar, instruction manual +++	Rechargeable battery 2 h +++	10×, 16× −7 to + 7 50–70 mm 0–11 mm Cobalt blue, green, conversion +++	Field, clinic Yes Yes +++
Portable slit lamp S150 (Medi-Works, Shanghai, China) (Mediworks, 2021) Slit lamp, attachment for phone	Ocular morbidities	Pricing not published +	Meant for researchers and ophthalmologists Anterior segment; 3.5 h ++	240 g; NR NR ++	Rechargeable batteries 6 h +++	6× NR NR 0–12 mm Cobalt blue +++	Field, clinic Yes Yes +++
SK-LS-1B portable slit lamp (Coburn Technologies, Inc. South Windsor, CT, USA) (Coburn Technologies Inc.) Slit lamp	Ocular morbidities	Pricing not published +	Meant for researchers and ophthalmologists Anterior segment; NR +++	835 g; 320 × 310 × 205 mm NR, optional iPhone adapter +++	Rechargeable batteries ≥4 h +++	10×, 16× −7 to + 7 49–75 mm 0.1, 0.2, 0.8, 1, 5, 12 mm Neutral density, red-free, cobalt blue +++	Field, clinic Yes Yes +++
**Device (manufacturer)** **Principle/method**	**Vitamin A biomarker**	**Pricing (estimated, list price from manufacturer website)**	**Technical requirements** **Sample site; time for full charge**	**Portability** **Included in kit**	**Power source** **Usage duration per charge**	**Dark adaptometers and other devices: Other attributes**	**Target setting** **Manufacturer support available** **Global availability**
RetEval (LKC Technologies, Gaithersburg, MD, USA) (Technologies, 2019) Non-mydriatic flash and flicker ERG/VEP device	Ocular morbidities	Pricing not published +	Meant for researchers and ophthalmologists Pupils, retina; 4 h full recharge time +++	240 g; 7 × 10 × 23 cm Storage case, charging stand +++	Battery-powered 8 h +++	Pupil measurements: 1.3–9 mm, <0.1 mm resolution, 28.3 Hz USB connectivity +++	Field, clinic Yes Yes +++
Mobile eye testing unit (Agrawal and Sahu, 2020) Vision drum, trial box, retinoscope, slit-lamp bio-microscope, applanation tonometer, and non-mydriatic fundus camera	Ocular morbidities: conjunctival xerosis with Bitot’s spot (X1B) or keratomalacia (X3B) (World Health, 2014)	Pricing not published +	Meant for researchers, optometrists, ophthalmologists Various parts of eye +++	Indicated to be mobile NR ++	Varies by device in kit ++	See (Agrawal and Sahu, 2020) +++	Field N/A; not commercially available No/custom ++
Scotopic Sensitivity Tester-1 (LKC Technologies, Gaithersburg, MD, USA) (Congdon et al., 1995, Congdon et al., 2000, Sanchez et al., 1997) Dark adaptometer	Visual score/threshold Pupillary score/threshold Pupillary dynamics Rod function	Pricing not published +	Meant for researchers and ophthalmologists Retina; binocular partial bleaching with camera flash (3433 cd-s/m^2^) +++	Hand-held N/A +++	NR NR +	Yellow-green LED light with wavelength at 572 nm, 12 intensity settings, calibrated with EG + G DR 2550 digital radiometer-photometer +++	Field, clinic Appears discontinued No +
Portable field dark adaptometer (custom) (Labrique et al., 2015, Palmer et al., 2015, Palmer et al., 2016) Dark adaptometer	Visual score/threshold Pupillary score/threshold Pupillary dynamics	Pricing not published +	Meant for researchers and ophthalmologists Retina; binocular partial bleaching with camera flash (>3400 cd-s/m^2^) +++	Portable: “Its size and weight allowed it to be carried long distances to areas unreachable by car.” Digital camera, a retinal bleaching flash, and a Ganzfeld light source inside a pair of light-obscuring goggles +++	Laptop-powered 10 tests per day ++	See (Labrique et al., 2015) Assess impaired pupillary responses to a graded series of Ganzfeld light stimuli applied within a pair of “darkroom” goggles with an embedded microcircuit design and regulated by a laptop-powered controller box ++	Field N/A; not commercially available No/custom? +
Emtech A meter V.01 (custom) (Mehta, 2018, Mehta, 2019b, Mehta, 2019a, Mehta and Mehta, 2018, Banerjee, 2019) Dark adaptometer	Dark adaptation; identify pictorial representation of objects at low light intensity	Pricing not published +	Meant for researchers and ophthalmologists Retina; NR +++	Handheld Electronic paper module, LCD to display test object, microSD card, keypad +++	NR NR +	Results output to microSD card +++	Field N/A; not commercially available No/custom? +
Custom-built portable field dark adaptometer (Steven and Wald, 1941, Wald, 1941) Dark adaptometer	Visual threshold Dark adaptation	Pricing not published +	Meant for researchers and ophthalmologists Retina; NR +++	8.4 kg; 21.6 × 21.6 × 29.2 cm; “approximate size and shape of a pocket lamp” Eyepiece, test unit, cord, cabinet +++	Three 2-volt discharge storage cells NR +++	Results in log units ++	Field, lab N/A; not commercially available No/custom? +
Reference method: Goldmann-Weekers dark adaptometer (Haag-Streit)	Visual threshold Dark adaptation	Not available for purchase +	Meant for researchers and ophthalmologists; some models require conversion table for a calibration error (Maggiano et al., 1978) Retina, with pupils dilated; NR Requires 60–120 min in a dark room +	Large size and complex; not portable N/A +	Requires external power source N/A +	Results: luminance in units of log microapostilbs, which requires conversion to the more contemporary unit of luminance, cd/m^2^ +	Clinic No Out of production and not available to order +

**Notes:** NR, not reported.

+++ = best.

++ = acceptable.

+ = not acceptable.

### Vitamin A deficiency biomarkers

In Table 2, we list definitions of VAD used across studies for a variety of biological sample types, from humans or cattle, including cows, calves, and bulls (Table 2). We also note which studies used particular definitions (e.g., VAD measured as RBP ≤ 0.70 µmol/L was measured by Hix et al. 2004). A previous review by Tanumihardjo (2016) has outlined the utility of biomarkers for vitamin A nutrition status (Tanumihardjo et al., 2016), which we adapted for Table 2. We outline the biomarkers of vitamin A as identified in our literature search below.

**Table 2:** Definitions of vitamin A deficiency, by sample type and device.

**Blood (whole, serum, plasma)**^a^			
**Biomarker**	**Type**^b^	**Device (studies using)**	**Deficiency or insufficiency definitions used**^c^
Retinol	Status	iCheck Fluoro (BioAnalyt)^d^ (Boateng et al., 2018, Elom et al., 2015, Ghaffari et al., 2019, Raila et al., 2017, Schweigert et al., 2011a, Bechir et al., 2012, Crump et al., 2017, Schweigert et al., 2011b, Whang et al., 2012, Zambo et al., 2012) Spectrophotometer model 450 (Sequoia-Turner) (Marinovic et al., 1997) CRAFTI (Craft Technologies) (Chaimongkol et al., 2011)	Severe/clinical deficiency: ≤0.35 µmol/L (10 µg/dL) (WHO, 2011) Low/subclinical deficiency: ≤0.70 µmol/L (20 µg/dL) (WHO, 2011) Insufficiency: ≤1.05 µmol/L (30 µg/dL) (de Pee and Dary, 2002)
RBP	Status	Custom REI (Hix et al., 2004) EE-µPAD (Lee et al., 2016) Tidbit (Lu and Erickson, 2017, Lu et al., 2017) ± HYPER filtration (Lu et al., 2018) Custom Ag-Ab reaction (Ciaiolo et al., 2015)	Deficiency: ≤0.70 µmol/L (Hix et al., 2004) Deficiency: <16.3 µg/mL^e^ (Lee et al., 2016) Deficiency: <14.7 µg/mL (correlated with retinol ≤ 0.70 µmol/L) (Lu et al., 2017) Not defined (Ciaiolo et al., 2015)
Beta-carotene	Not defined (indicator of recent dietary intake)	iCheck Carotene (BioAnalyt)^f^ (Ghaffari et al., 2019, Hye et al., 2020, Klein et al., 2013, Livingston et al., 2020, Meinke et al., 2016, Raila et al., 2012, Madureira et al., 2020)	Humans: No official cut-off defined (von Lintig, 2020) Cattle (Klein et al., 2013, De Ondarza and al., 2009, Schweigert and Immig, 2007): Deficient: 0.6–1.5 mg/L or < 1.5 mg/L Marginal: ≥1.5 mg/L to < 3.5 mg/L Optimal: ≥3.5 mg/L
**Milk**			
**Biomarker**	**Type**^b^	**Device**	**Deficiency or insufficiency definitions used**^c^
Retinol	Status, exposure	iCheck Fluoro (BioAnalyt)^d^ (Jans et al., 2018, Abebe et al., 2019, Engle-Stone et al., 2014, Schweigert et al., 2011a, Schweigert et al., 2011b, Bechir et al., 2012, Crump et al., 2017)	Humans: Inadequate: <1.05 µmol/L (Blaner, 2020) *or* milk fat < 8 µg/g (Blaner, 2020) Cattle: not defined
Beta carotene	Not defined (indicator of recent dietary intake)	iCheck Carotene (BioAnalyt)^d^ (no studies)	Humans: not defined Cattle: not defined
**Eyes**			
**Biomarker**	**Type**^b^	**Device (studies using)**	**Deficiency or insufficiency definitions used**^c^
Visual score/threshold	Function	Scotopic sensitivity hand-held illuminator (LKC Technologies, Inc.) (Congdon et al., 1995, Sanchez et al., 1997, Reilly et al., 2006) EmTech A meter V.01^g^ (Mehta, 2018) Portable visual adaptometer (Wald, 1941, Steven and Wald, 1941) Scotopic Sensitivity Tester-1^TM^ (SST-1) (Peters et al., 2000)	Abnormal: ≥stimulus #10 (Congdon et al., 1995) ≥-3.76 log cd/m^2 h^ (Congdon et al., 1995) Highly abnormal: ≥stimulus #11 ≥-3.39 log cd/m^2^ (Congdon et al., 1995) A decrease of ≥ 0.3 log units after administration of vitamin A supplementation (Wald, 1941, Steven and Wald, 1941)
Dark adaptation: pupillary score/responsiveness [lowest light intensity that stimulated percentage relative change in pupil diameter (Labrique et al., 2015)]	Function	Scotopic sensitivity hand-held illuminator (LKC Technologies, Inc.) (Congdon et al., 1995, Sanchez et al., 1997, Peters et al., 2000) Portable field dark adaptometer (PFDA) or digital pupillometer (Labrique et al., 2015, Palmer et al., 2015, Palmer et al., 2016)	Normal: ≥-1.24 log cd/m^2^ (Congdon and West, 2002) Abnormal: ≥stimulus #9 (Congdon et al., 1995) ≥-0.575 log cd/m^2^ (Congdon et al., 1995) i.e.,≥20% (Labrique et al., 2015) ≥-1.11 log cd/m^2^ (Congdon et al., 2000) ≥-0.9 log cd/m^2^ (Palmer et al., 2016) ≥15% relative change in diameter (Labrique et al., 2015) ≥10% contraction in pupil size (Palmer et al., 2016) Threshold: ≥15 cd/m^2^ (Khan et al., 2019)
Pupillary dynamics [i.e., response time: absolute value of difference in frame numbers from pre- to post-stimulus divided by number of frames per second (Labrique et al., 2015)]	Function	Portable field dark adaptometer (PFDA) or digital pupillometer (Labrique et al., 2015, Palmer et al., 2015, Palmer et al., 2016)	No official cut-off defined
Rod function [dark-adapted rod full-field electro-retinogram responses (Peters et al., 2000)]	Function	Scotopic Sensitivity Tester-1^TM^ (SST-1) (Peters et al., 2000)	No official cut-off defined
Ocular morbidities	Function	Mobile eye unit (comprised of vision drum, trial box, retinoscope, slit-lamp bio-microscope, applanation tonometer, non-mydriatic fundus camera) (Agrawal and Sahu, 2020) Heine HSL-100 biomicroscope equipped with portable slit lamp (Melo et al., 2004)	Night blindness, conjunctival xerosis with Bitot’s spots (X1B), keratomalacia (X3B), ocular lesions (Agrawal and Sahu, 2020) (stages as designated by World Health Organization grading system (World Health, 2014) Ocular lesions (Melo et al., 2004)

***Notes:****Ag-Ab, antigen*–*antibody; EE-µPAD, electronics enabled microfluidic paper-based analytical device; HYPER, High-yield paper-based quantitative blood separation system; RBP, retinol-binding protein; REI, rapid enzyme immunoassay*.

h cd/m^2^ is the SI unit of luminance (Congdon et al., 2000).

a Adapted from reference (Tanumihardjo et al., 2016).

b Whereas serum or plasma is required to measure circulating vitamin A, some devices can use whole blood as the sample input.

c Defined by global standards (e.g., World Health Organization) or by study authors.

d An earlier version of this device is referenced as iCheck Ret 435–1 (Bechir et al., 2012).

e Correlated with retinol ≤ 0.70 µmol/L, when sandwich ELISA is used for RBP measurement (Erhardt et al., 2004).

f An earlier version of this device is referenced as iCheck Ret 515–2 (Bechir et al., 2012).

g Device also referenced as “dark adaptometer” (Banerjee, 2019) or “In-Direct method and system for Vitamin A deficiency detection” (Mehta, 2019b, Mehta, 2019a, Mehta and Mehta, 2018).

Vitamin A liver concentration (µmol vitamin A/g liver) is the gold standard for vitamin A status but requires invasive techniques such as biopsy to be measured (Tanumihardjo et al., 2016). Sampling blood enables the quantification of serum retinol, serum retinol-binding protein (RBP), or provitamin A in the form of beta-carotene; however, each measure has trade-offs. Serum retinol reflects liver stores only at extremes of deficiency (≤0.07 µmol/g liver) or elevation (>1.05 µmol/g liver) (WHO, 2011), because serum retinol is homeostatically regulated by the body. The World Health Organization defines VAD as serum retinol ≤ 0.70 µmol/L (WHO, 2011). RBP is commonly assumed to have a 1:1 ratio with serum retinol, and therefore the same cut-offs are sometimes used for both retinol and RBP. However, this ratio can be affected by the extent of VAD, zinc deficiency, acute phase response, protein-energy malnutrition, liver disease, acutely stressful situations, high fever, antibiotic use, or obesity (Tanumihardjo et al., 2016, de Pee and Dary, 2002). Therefore, previous studies have proposed other deficiency cut-offs, such as 0.69 µmol/L (Semba et al., 2002) or 0.83 µmol/L (Engle-Stone et al., 2011, Gorstein et al., 2008). Recently, the Global Alliance for Vitamin A has recommended analysis of a subsample by HPLC to confirm the cut-off point for VAD; furthermore, given the acute phase response, inflammation markers such as C-reactive protein and alpha-1-acid-glycoprotein must also be measured (Global Alliance for Vitamin A, 2019).

Beta carotene is one of several dietary provitamin A carotenoids, a plant-derived form of vitamin A. The body converts dietary provitamin A carotenoids into retinol with the following conversion factors: 1 µg retinol activity equivalent (RAE) equals 1 retinol equivalent (RE), 1 µg retinol, 2 µg β-carotene in oil, 12 µg β-carotene in mixed foods, or 24 (12–26) µg other provitamin A carotenoids in mixed foods (Institute of Medicine, 2001, Combs and McClung, 2017, Blaner, 2020). The conversion efficiency ratio of beta carotene to RAE is still debated. For example, the European Food Safety Authority suggests that the conversion is 6:1 rather than 12:1 (EFSA Panel on Dietetic Products, Nutrition Allergies, 2015). Carotenoids can be measured in blood, milk, or skin, and several studies have found a positive association of skin carotenoid concentrations with serum or plasma carotenoid status (Zidichouski et al., 2009, Aguilar et al., 2014, Morgan et al., 2019, Hayashi et al., 2020). However, a consensus has not been reached regarding a conversion factor or how the measurements equate to vitamin A status (von Lintig, 2020). Because carotenoids tend to reflect recent dietary intake rather than long-term status, recommended serum carotenoid deficiency cut-offs have not been established in humans (von Lintig, 2020). Deficiency in β-carotene in cow’s blood has been defined as 0.6–1.5 mg/L (Klein et al., 2013, De Ondarza and Al, 2009, Schweigert and Immig, 2007).

In breast milk, retinol may be measured to estimate both the maternal vitamin A status and intake, and the infant intake of vitamin A (Engle-Stone et al., 2014, Tanumihardjo et al., 2016). Additionally, breast milk retinol measurement is influenced by the stage of lactation, time of day, “fullness” of the breast, feeding status if milk from both breasts is analyzed, and whether the milk is hindmilk compared with foremilk (Tanumihardjo et al., 2016). VAD is defined as a milk retinol concentration ≤ 1.05 µmol/L, or ≤ 8 µg/g milk fat (Tanumihardjo et al., 2016). In cows, milk β-carotene levels are often measured and linked to bovine fertility and health.

Because of vitamin A’s role in in producing rhodopsin, the visual pigment of rods in the eyes, VAD can cause ocular manifestations resulting in poor vision (World Health, 2014). These include night blindness, conjunctival xerosis, Bitot’s spots, corneal xerosis, and keratomalacia. Impaired adaption to the dark is among the first symptoms of VAD, and it can be used as a screening tool (World Health, 2014). Tests such as pupillary and visual thresholds can assess dark adaptation by determining the lowest-intensity level of light required to cause pupillary dilation or to visualize an image (World Health, 2014, Labrique et al., 2015).

### Comparison studies

From 3230 studies (after de-duplication), we identified 18 studies (19 reports) comparing nine portable methods/devices (index 1) to a reference standard method (Fig. 1); we were unable to retrieve an additional two reports (Craft, 2005, Fujita, 2007). No studies compared two portable methods (i.e., index 1 vs. index 2). Thirteen studies (15 reports) measured human or cattle blood samples (BioAnalyt, 2020, Chaimongkol et al., 2011, Ciaiolo et al., 2015, Elom et al., 2015, Ghaffari et al., 2019, Hix et al., 2004, Hix et al., 2006, Lee et al., 2016, Lu et al., 2017, Lu et al., 2018, Raila et al., 2012, Raila et al., 2017, Schweigert et al., 2011a, Chaimongkol et al., 2008, Lu and Erickson, 2017); four studies measured human or cattle milk samples (Schweigert et al., 2011b, Schweigert et al., 2011a, Engle-Stone et al., 2014, Abebe et al., 2019); and one study measured eye function (Peters et al., 2000). Study details are listed in Table 3. We also re-analyzed the data presented in supplemental Tables S1 and S2 in one publication to calculate the descriptive statistics for plasma and whole blood retinol in samples analyzed by HPLC and iCheck Fluoro (Raila et al., 2017).

**Table 3:** Description of included studies comparing a portable method against a reference standard method.

Author Year	Device	Manufacturer	Sample tested Biomarker	Study population	Test location (field/laboratory, country)	Reference method	Ref.
Chaimongkol 2011	CRAFTi	Eurofin Craft Technologies	Serum Retinol	Study cohorts	Thailand	HPLC	(Chaimongkol et al., 2011)
Chaimongkol 2008^a^	CRAFTi	Eurofin Craft Technologies	Serum Retinol	Study cohorts	Thailand	HPLC	(Chaimongkol et al., 2008)
Ciaiolo 2015	Custom Ab-Ag reaction	Custom	Serum, RBP	Patients	Italy	Nephelometry	(Ciaiolo et al., 2015)
Lee 2016	EE-µPAD	Custom	Serum RBP	Commercial (ProMedDx)	USA	ELISA	(Lee et al., 2016)
BioAnalyt report (year: NR)	iCheck Carotene	BioAnalyt	Plasma	Dairy cows and calves	NR	HPLC	(BioAnalyt, NR)
Raila 2012	iCheck Carotene	BioAnalyt	Whole blood or plasma Beta-carotene	Holstein-Friesian cows, local farm	Germany, Ireland, France	HPLC	(Raila et al., 2012)
Ghaffari 2019	iCheck Carotene	BioAnalyt	Plasma Beta-carotene	Holstein cows and calves from institutional farms	Germany	HPLC	(Ghaffari et al., 2019)
	iCheck Fluoro	BioAnalyt	Whole blood Retinol				
Raila 2017	iCheck Fluoro	BioAnalyt	Whole blood, serum Retinol	Dairy cows and bulls, institutional farms	Germany, Japan	HPLC	(Raila et al., 2017)
Schweigert 2011a	iCheck Fluoro	BioAnalyt	Milk Retinol	Study cohorts and local cows	Germany	HPLC	(Schweigert et al., 2011b)
Schweigert 2011b^a^	iCheck Fluoro	BioAnalyt	Plasma or milk Retinol	Study cohorts	Low resource setting	HPLC	(Schweigert et al., 2011a)
Abebe 2019	ICheck Fluoro	BioAnalyt	Milk Retinol	Study cohorts	Ethiopia	HPLC	(Abebe et al., 2019)
Elom 2015	iCheck Fluoro	BioAnalyt	Serum Retinol	Study cohorts	Morocco	HPLC	(Elom et al., 2015)
Engle-Stone 2014	iCheck Fluoro	BioAnalyt	Milk Retinol	Study cohorts	Cameroon	HPLC	(Engle-Stone et al., 2014)
Hix 2004	RBP-EIA	Scimedx Corp	Serum RBP	Study cohorts, commercial	Papua New Guinea, Nicaragua	HPLC	(Hix et al., 2004)
	RID	The Binding Site		Study cohorts	Nicaragua	HPLC	
Hix 2006	RBP-EIA	Scimedx Corp	Serum RBP	Study cohorts	Cambodia	HPLC	(Hix et al., 2006)
Peters 2000	SST-1	LKC Technologies	Eyes Dark-adapted final thresholds; rod function	Patients of Retina Foundation of the Southwest	USA	Goldmann-Weekers Dark Adaptometer	(Peters et al., 2000)
Lu 2017	Tidbit, ± HYPER filtration system	Custom	Serum RBP	Commercial (Research Blood Components LLC)	USA	ELISA	(Lu and Erickson, 2017, Lu et al., 2017, Lu et al., 2018)

*Notes: Ag-Ab, antigen*–*antibody; EE-µPAD, electronics enabled microfluidic paper-based analytical device; EIA, enzyme immunoassay; HYPER, high-yield paper-based quantitative blood separation system; RBP, retinol-binding protein; RID, radial immunodiffusion assay; SST-1, Scotopic Sensitivity Tester-1*.

^a^ Meeting abstract, therefore some details are not reported.

^b^ RID may be considered a second index test, because it is not a reference standard; however, in the study, only the first index test, RBP-EIA was the assay undergoing development and validation.

We also identified many studies that used a portable device for assaying samples but did not compare the results to those of a reference method and instead cited previous validation studies. Although the devices used are catalogued and described (Table 1a, Table 1b), these studies are not further detailed in this review.

Study populations were mostly from the US and Germany, in addition to Thailand, Italy, France Ireland, Japan, Ethiopia, Morocco, Cameroon, Papua New Guinea, Nicaragua, Cambodia, and Oman. Portable fluorometers, photometers, enzyme-based assays or immunoassays, microfluidics-based approaches, and a dark adaptometer for eye function were assessed and compared with their respective reference standards.

Table 4 compares the stated performance criteria described by the device manufacturers’ websites to reporting from individual studies using the devices, according to the WHO **A**ffordable, **S**ensitive, **S**pecific, **U**ser-friendly, **R**apid and **r**obust, **E**quipment-free and **D**eliverable to end-users (ASSURED) criteria for diagnostic tests in resource-limited settings (Kosack et al., 2017). Only the iCheck Fluoro and iCheck Carotene stated performance criteria on the BioAnalyt website and are included in this table. No studies described the cost of the devices. Most devices did not have published cost information, aside from some of the slit lamps showing list prices. Other devices described by the studies were developed as proofs of concept and are either not on the market or are on the market, but lacking performance criteria on the manufacturer’s website.

**Table 4:** Assessment of devices against manufacturer-reported performance, according to ASSURED* criteria. (Ghaffari et al., 2019, Raila et al., 2017, Schweigert et al., 2011b, Schweigert et al., 2011a, Abebe et al., 2019, Elom et al., 2015, Engle-Stone et al., 2015, Raila et al., 2012, Ghaffari et al., 2019.)

We note that the study by Ghaffari et al. (2019) reported both measurements of retinol in whole blood and beta carotene in plasma, but only directly references the iCheck Fluoro device and not the iCheck Carotene (Ghaffari et al., 2019). Whereas the iCheck Carotene requires 0.4 mL of sample, the iCheck Fluoro requires 0.5 mL; the authors stated using only 0.5 mL of sample.

Additionally, the manufacturer (BioAnalyt) lists only “colostrum, cattle whole blood, and serum” as appropriate sample types for the iCheck Carotene; however all studies using the iCheck Carotene, including a BioAnalyt report, also analyzed plasma for beta carotene content (BioAnalyt, 2020, Ghaffari et al., 2019, Raila et al., 2012).

A major gap across all devices is the lack of reporting on sensitivity and specificity compared with a reference standard method.

Most studies compared a portable device to a reference method. Table 5a, Table 5b, Table 5c, Table 5d, Table 5e, Table 5f, Table 5g show the performance of these devices against their reference standards for measuring vitamin A and VAD. Additional analyses conducted with other index (e.g., index 2) tests are described in the text.

**Table 5a:** Portable fluorometers: device performance in human blood samples.

**Portable device vs. reference**	**iCheck Fluoro vs. HPLC**	**iCheck Fluoro vs. HPLC**	**CRAFTi vs. HPLC**	**CRAFTi vs. HPLC**
Vitamin A biomarker	Retinol^a^	Vitamin A^a^	Retinol^a^	Retinol^a^
Sample type	Plasma	Serum	Serum	Serum
Study population	89 children	56 samples	38 women	75 women, 143 children
Concentration difference	MD: 0 min: 1.9 µg/L ± 23.2 MD: 15 min: −8.0 µg/L + 22.7	NR	MD: −0.07	MD: −0.07
Correlation coefficient	0 min: 0.98 15 min: 0.98	NR	NR	0.77
R^2^	NR	>0.95	NR	NR
Regression equation	NR	NR	Slope = 0.81	NR
Operational range	NR	NR	NR	0.5–1.5 µmol/L
VAD or VAI (%), index vs. ref	Not defined: N = 2/89 vs. NR	NR	NR	<0.7 µmol/L: 9.2% vs. 2.8% <1.05 µmol/L: 49.5% vs. 43.6%
**Precision**				
Sensitivity	NR	NR	NR	VAD ≤ 0.7 µmol/L: 66.7% VAD ≤ 1.05 µmol/L: 85.3%
Specificity	NR	NR	NR	VAD ≤ 0.7 µmol/L: 92.4% VAD ≤ 1.05 µmol/L: 78.0%
Intra-assay %CV	NR	2.5–6.4 %^b^	Agreement noted but not quantified	3.97% vs. 3.45%
Inter-assay %CV	NR			NR
Inter-observer %CV	NR			NR
Bland Altman analysis comments	No commentary. At 0 min, 3 values fell outside 2 SDs. At 15 min, 4 values fell outside 2 SDs	NR	No systematic bias	No systematic bias; most values within ± 0.5 with normally distributed serum retinol values
Reference	(Elom et al., 2015)	(Schweigert et al., 2011a)^c^	(Chaimongkol et al., 2008)^c^	(Chaimongkol et al., 2011)

***Notes:****MD, mean difference; NR, not reported; RE, retinol equivalents defined as the sum of retinol and retinyl esters, equal to 3.3 International Units (IU) of vitamin A or as* 1 µ*g (units reported by manufacturer—however, retinol activity equivalents (RAE) are the preferred unit for reporting (*Institute of medicine, 2001*); SD, standard deviation; VAD, vitamin A deficiency; VAI, vitamin A insufficiency*.

a Units: µg/L or µmol/L.

b Specific %CVs not distinguished.

c Study abstract, lacking some details.

**Table 5b:** Portable fluorometers: device performance in human and bovine milk samples.

	**Human milk**			**Bovine milk**
**Portable device vs. reference**	**iCheck Fluoro vs. HPLC**	**iCheck Fluoro vs. HPLC**	**iCheck Fluoro vs. HPLC**	**iCheck Fluoro vs. HPLC**
Vitamin A biomarker	Retinol^a^, milk fat^b^	Retinol^a^, milk fat^b^	Retinol^a^	Retinol^a^
Study population	104 women	75 women, 154 samples	1 woman, 16 samples	21 cows
Concentration difference	MD: 0.01 µmol/L, 0.03 µg/g fat	MD: −0.83 ± 0.14 µmol/L, −5.6 ± 0.7 µg/g fat	Expressed milk, MD: 103% ± 13	Expressed milk, MD: 105% ± 9 Powdered milk (n = 5), MD: 144% ± 15 Liquid whole milk (n = 5), MD: 118% ± 13 Liquid skim milk (n = 4), MD: 95% ± 10
Correlation coefficient	0.57_unadj_, 0.59_adj_^c^	0.85_unadj_, 0.79_adj_^c^	NR	NR
R^2^	0.32_unadj_, 0.35_adj_^c^	0.72_unadj_, 0.62_adj_^c^	NR	NR
Regression equation	NR	NR	NR	NR
Operational range	50–3000 µg RE/L	50–3000 µg RE/L	50–3000 µg RE/L^d^	NR
VAD or VAI (%), index vs. ref	<1.05 µmol/L: 87% vs. 76% <8 µg/g fat%: 89% vs. 81%	<1.05 µmol/L: 3.9% vs. 2.60% <8 µg/g fat %: 0% vs. 2%	NR	NR
**Precision**				
Sensitivity	NR	Too few VAD cases to examine	NR	NR
Specificity	NR	Too few VAD cases to examine	NR	NR
Intra-assay %CV	1.1% vs. 1.5–1.6%	0.6 %^e^	NR	NR
Inter-assay %CV	NR		NR	NR
Inter-observer %CV	NR		NR	NR
Bland Altman analysis comments	Used to present mean difference between measurements; mean difference not significantly different from zero	Plotted but no conclusion drawn; appears to show 8 values outside of 2 SDs (µmol/L retinol) and 8 values outside of 2 SDs (µg/g fat)	NR	NR
Reference	(Abebe et al., 2019)	(Engle-Stone et al., 2014)	(Schweigert et al., 2011b)	(Schweigert et al., 2011b)

***Notes:****MD, mean difference; NR, not reported; RE, retinol equivalents defined as the sum of retinol and retinyl esters, equal to 3.3 International Units (IU) of vitamin A or as* 1 µ*g (units reported by manufacturer—however, retinol activity equivalents (RAE) are the preferred unit for reporting (*Institute of medicine, 2001*); VAD, vitamin A deficiency; VAI, vitamin A insufficiency*.

a Units: µmol/L or µg RE/L.

b Units: µg/g fat%.

c Adjusted for breast milk fat content.

d Not reported but based on previous studies using same device.

e Specific %CVs not distinguished.

**Table 5c:** Portable immunoassays: device performance in human blood.

**Portable device vs. reference**	**RBP-REI vs. HPLC**^a^		**RBP-REI vs. HPLC**^a^	**RID vs. HPLC**^a^	**Immunoassay vs. nephelometry**	
Vitamin A biomarker	RBP^b^	RBP^b^	RBP^b^	RBP^b^	RBP^b^	
Sample type	Serum	Serum	Serum	Serum	Serum	
Study population	24 children	70 mothers and children	359 children	40 mothers and children	2 healthy adults (Serum A, B)	
Concentration difference	MD: 0.22 µmol/L	NR	NR	NR	Index (dilution):	
					Serum A: (1:10): + (1:100): - (1:1000): - (1:10000): -	Serum B: (1:10): ++ (1:100): - (1:1000): - (1:10000): -

Ref: Serum A: 46 mg/L	Ref: Serum B: 42 mg/L
Correlation coefficient	0.93	0.91	0.89	0.84	NR	
R^2^	0.86	0.82	0.79	0.71	NR	
Regression equation	y = 0.95x + 0.36	y = 0.62x + 0.32	y = 0.65x + 0.27	NR	NR	
Operational range	10–40 µg RBP/mL	10–40 µg RBP/mL	10–40 µg RBP/mL^c^	NR	Immune precipitates: Neg: - 2 mg/L: + 10 mg/L: ++ 100 mg/L: +++ 1000–10000 mg/L: ++++	
VAD or VAI (%), index vs. ref	≤0.70 µmol/L: 32% vs. 36%	NR	<0.35 µmol/L: 0.6% vs. 2.2% ≤0.70 µmol/L: 20.9% vs. 22.3%	NR	NR	
**Precision**						
Sensitivity	NR	NR	70%	NR	“Good” (“can detect presence of [RBP] at concentration of few µg/mL”)	
Specificity	NR	NR	93.2%	NR	NR	
Intra-assay %CV	6.7 %^d^	NR	NR	NR	NR	
Inter-assay %CV	8.9 %^d^	NR	NR	NR	NR	
Inter-observer %CV	13.0 %^d^	NR	NR	NR	NR	
Bland Altman analysis comments	NR	NR	NR	NR	NR	
Reference	(Hix et al., 2004)		(Hix et al., 2006)	(Hix et al., 2004)	(Ciaiolo et al., 2015)	

***Notes:*** MD*, mean difference; NR, not reported; RBP, retinol binding protein; RE, retinol equivalents defined as the sum of retinol and retinyl esters, equal to 3.3 International Units (IU) of vitamin A or as* 1 µ*g (units reported by manufacturer—however, retinol activity equivalents (RAE) are the preferred unit for reporting (*Institute of Medicine, 2001*); REI, rapid enzyme immunoassay; VAD, vitamin A deficiency; VAI, vitamin A insufficiency*.

a Reference analyte is retinol.

b Units: µmol/L or µg RE/L.

c Not reported but based on previous studies using same device.

d Reported from separate analysis among unknown total # of samples (“5 adult volunteers and a commercially available source”) analyzing device performance, without reference to HPLC.

**Table 5d:** Portable microfluidics-based methods: device performance in human blood.

**Portable device vs. reference**	**EE-µPAD, vs. ELISA**	**Tidbit with HYPER platform, vs. ELISA**^a^	**Tidbit without HYPER platform, vs. ELISA**
Vitamin A biomarker^f^	RBP^b^	RBP^b^	RBP^b^
Sample type	Whole blood	Whole blood	Serum
Study population	95 adults (commercial)	12 adults	43 adults (commercial)
Concentration difference	NR	NR	NR
Correlation coefficient	NR	NR	0.75
R^2^ (index, unless specified)	NR	Index: 0.81 vs. ref: >0.99	0.56
Regression equation	NR	Slope = 0.99	Slope = 0.97
RMSE, index vs. ref	NR	3.75 vs. 1.3 µg/mL	4.34 µg/mL vs. NR
Operational range	∼10–70 µg/mL (graph)	∼5–20 µg/mL (graph)	2.2–20 µg/mL (0.10–0.95 µmol/L)
VAD or VAI (%), index vs. ref	<16.3 µg/mL: AUC = 0.7139 vs. 17.2%	NR	<14.7 µg/mL (≤0.70 µmol/L): NR vs. 9.3%
**Precision**			
Sensitivity	75% at MFR cutoff, 0.831	NR	100%
Specificity	62.3% at MFR cutoff, 0.831	NR	100%
Intra-assay %CV	10.8% vs. 3.9%	20.3% deviation per test strip, recommend taking average of 3 test strips	NR
Inter-assay %CV	NR	NR	NR
Inter-observer %CV	NR	NR	NR
Bland Altman analysis comments	NR	NR	Bias at −0.05 µg/mL (-2.3 nmol/L)
Reference	(Lee et al., 2016)	(Lu and Erickson, 2017, Lu et al., 2017, Lu et al., 2018)	(Lu and Erickson, 2017, Lu et al., 2017)

***Notes:****MD, mean* difference*; MFR, multi-faceted ratio* i.e.*, the ratio of the light transmission in the test area to that in the background control area, calculated for RBP for each sample repeat. NR, not reported; RE, retinol equivalents defined as the sum of retinol and retinyl esters, equal to 3.3 International Units (IU) of vitamin A or as* 1 µ*g (units reported by manufacturer—however, retinol activity equivalents (RAE) are the preferred unit for reporting (*Institute of medicine, 2001*); RMSE, root mean squared error; VAD, vitamin A deficiency; VAI, vitamin A insufficiency*.

a Reference ELISA utilized samples that were filtered using HYPER system.

b Units: µg/mL, mg/L, or µmol/L.

**Table 5e:** Other portable devices: device performance in for assessing eye function (vision).

	**Eyes**
	**Index 1**^a^**vs. reference**^b^**^,^**^c^
**Validation of portable device**	**SST-1 vs. Goldmann-Weekers dark adaptometer**^a^
Vitamin A biomarker	Dark adaptation final threshold^b^
Study population	87 patients^c^ and 24 healthy children and adults
Concentration difference	NR
Correlation coefficient	0.88 (adjusted for ceiling effect)
R^2^ (index, unless specified)	0.77
Regression equation	“intercept close to zero”
Operational range	0–30 dB stimulus intensity range (0–3 log units)
VAD or VAI (%), index vs. ref	Elevated final thresholds: 75% vs. 82%
**Precision**	
Sensitivity	Final threshold elevated: 74.7%
Specificity	NR
Intra-assay %CV	NR
Inter-assay %CV	NR
Inter-observer %CV	NR
Bland Altman analysis comments	NR
Reference	(Peters et al., 2000)

***Notes:*** MD*, mean difference; MFR, multi-faceted ratio* i.e.*, the ratio of the light transmission in the test area to that in the background control area, calculated for RBP for each sample repeat. NR, not reported; RE, retinol equivalents defined as the sum of retinol and retinyl esters, equal to 3.3 International Units (IU) of vitamin A or as* 1 µ*g (units reported by manufacturer—however, retinol activity equivalents (RAE) are the preferred unit for reporting (*Institute of medicine, 2001*); SST-1, Scotopic Sensitivity Tester-1; VAD, vitamin A deficiency; VAI, vitamin A insufficiency*.

a Reference analyte is dark adaptation final threshold.

b Units: log units.

c Patients had retinal degeneration with mild to severe loss of rod function from full-field ERG results.

**Table 5f:** Portable fluorometers: device performance in bovine blood samples.

**Portable device vs. reference**	**iCheck Fluoro vs. HPLC**^a^		**iCheck Fluoro vs. HPLC**^a^	**iCheck Fluoro vs. HPLC**^a^		**iCheck Fluoro vs. HPLC**^a^		**iCheck Fluoro vs. HPLC**^b^	
Vitamin A biomarker	Vitamin A^c^	Vitamin A^c^	Retinol^d^	Vitamin A^c^	Vitamin A^c^	Retinol^d^	Retinol^d^	Retinol^d^	Retinol^d^
Sample type	Whole blood	Whole blood	Whole blood	Plasma	Plasma	Plasma	Plasma	Serum	Serum
Study population	28 cows	11 calves	10 cows	28 cows	11 calves	40 cows	92 bulls	29 cows	32 black cattle
Concentration difference	Range: 184–336	(see note)^e^	MD: −0.013 ± -0.020	MD: 19.3	MD: 26.5	MD: 0.01		MD: 0.00	
Correlation coefficient	0.78	0.90	0.92	0.88	0.96	0.94		0.93	
R^2^	0.61	0.81	0.84	0.77	0.92	0.88		g0.87	
	0.88			0.94					
Regression equation	y = 0.77 + 11.26		NR	y = 1.18x − 72.64	y = 0.80x + 1.32	NR		NR	
				y = 1.03–30.11					
Operational range	NR		NR	NR		NR		NR	
VAD or VAI (%)	NR	NR	NR	NR	NR	NR	NR	NR	NR
**Precision**									
Sensitivity	NR	NR	States test is sensitive and specific but does not quantify these	NR	NR	States test is sensitive and specific but does not quantify these	States test is sensitive and specific but does not quantify these	States test is sensitive and specific but does not quantify these	States test is sensitive and specific but does not quantify these
Specificity	NR	NR		NR	NR				
Intra-assay %CV	NR	NR	NR	NR	NR	2.3% vs. 5.3 %^f^		2.1% vs. 3.3 %^f^	
Inter-assay %CV	NR	NR	NR	NR	NR				
Inter-observer %CV	NR	NR	NR	NR	NR	NR	NR	NR	NR
Bland-Altman analysis comments	Good level of agreement and no systematic error. 5% of total values fell outside 95% acceptability limits		Good level of agreement and no systematic error; 4% of total values fell outside the 95% acceptability limits	Good level of agreement and no systematic error. 1 value fell outside of 95% acceptability limits	Good level of agreement and no systematic error. 1 value fell outside of 95% acceptability limits	Good level of agreement and no systematic error. 4% of values fell outside the 95% acceptability limits			
Reference	(Ghaffari et al., 2019)		(Raila et al., 2017)	(Ghaffari et al., 2019)		(Raila et al., 2017)			

***Notes:****MD, mean difference; NR, not reported; RE, retinol equivalents defined as the sum of retinol and retinyl esters, equal to 3.3 International Units (IU) of vitamin A or as* 1 µ*g (units reported by manufacturer—however, retinol activity equivalents (RAE) are the preferred unit for reporting (*Institute of medicine, 2001*); VAD, vitamin A deficiency; VAI, vitamin A insufficiency*.

a Reference sample is in plasma.

b Reference sample is serum.

c Units: µg RE/L.

d Units: µmol/L.

e Reported value from this study appears to be a repeated value for cow whole blood beta carotene content given as 2.09–8.15 mg/L, instead of the calf whole blood vitamin A reported in µg RE/L.

f Values appear to be an average of intra- and inter-assay %CV.

**Table 5g:** Portable photometers: device performance in bovine blood samples.

**Portable device vs. reference**	**iCheck Carotene vs. HPLC**^a^	**iCheck Carotene vs. HPLC**^a^	**iCheck Carotene vs. HPLC**^a^	**iCheck Carotene vs. HPLC**^a^	**iCheck Carotene vs. HPLC**^a^	**iCheck Carotene vs. HPLC**^a^	**iCheck Carotene vs. HPLC**^a^
Vitamin A biomarker	β-carotene^b^	β-carotene^b^	β-carotene^b^	β-carotene^b^	β-carotene^b^	β-carotene^b^	β-carotene^b^
Sample type	Whole blood	Whole blood	Whole blood	Plasma	Plasma	Plasma	Plasma
Study population	28 cows	11 calves	23 cows	28 cows	11 calves	NR, cows and calves	166 cows
Concentration difference	NR	NR	MD: 0.21	MD: −0.29	MD: 0.02	NR	MD: 0.26
Correlation coefficient	0.98	0.98	0.99	0.97	0.98	NR	0.99
R^2^	0.97	0.96	0.99	0.93	0.96^c^	0.97	0.98
	0.99^c^			0.98^d^			
Regression equation	y = 1.01x + 0.17^c^		NR	y = 0.88x + 0.31^e^ y = 0.97x + 0.40^e^	y = 1.05x + 0.04^e^ y = 0.90x + 0.04^e^	y = 0.90x + 0.17	y = 0.98x + 0.31
Operational range	NR		0.4–18 mg/L	NR		∼0–9 mg/L (graph)	0.4–18 mg/L
VAD (%)	NR	NR	NR	NR	NR	NR	NR
**Precision**							
Sensitivity	NR	NR	NR	NR	NR	NR	NR
Specificity	NR	NR	NR	NR	NR	NR	NR
Intra-assay %CV	NR	NR	3.5% vs. 2.3 %^f^	NR	NR	NR	3.5% vs. 2.3 %^f^
Inter-assay %CV	NR	NR	NR	NR	NR	NR	NR
Inter-observer %CV	NR	NR	NR	NR	NR	NR	NR
Bland Altman analysis comments	A good level of agreement and no systematic error for β-carotene and vitamin A; “only 5% of the differences in measured values fell outside the 95% acceptability limits for β-carotene in dairy cows”		Systematic error did not occur between methods: 4% of differences outside 95% limits	A good level of agreement and no systematic error for β-carotene and vitamin A; “only 5% of the differences in measured values fell outside the 95% acceptability limits for β-carotene in dairy cows”		Graph presented, no comment (appears to have good agreement)	Systematic error did not occur between methods: 4% of differences outside 95% limits
Reference	(Ghaffari et al., 2019)		(Raila et al., 2012)	(Ghaffari et al., 2019)		(BioAnalyt, NR)	(Raila et al., 2012)

**Notes:** MD, mean difference; NR, not reported; RE, retinol equivalents defined as the sum of retinol and retinyl esters, equal to 3.3 International Units (IU) of vitamin A or as 1 µg (units reported by manufacturer—however, retinol activity equivalents (RAE) are the preferred unit for reporting (Institute of Medicine, 2001).

a Reference sample is in plasma.

b Units: mg/L.

c Average from reference analyses done in Germany and Switzerland.

d Reference analysis done in Germany.

e Reference analysis done in Switzerland.

f Appears to represent the average CV for both whole blood and plasma samples.

In human blood samples, both the iCheck Fluoro and the CRAFTi portable fluorometers were used to measure retinol (Table 5a). The iCheck Fluoro studies showed a high correlation (0.98) and an R-squared values over 0.95 with respect to HPLC. Both CRAFTi studies found a mean difference in serum retinol of −0.07 µmol/L, and the 2011 study found moderate sensitivity and specificity in identifying VAD at either ≤ 0.70 µmol/L or ≤ 1.05 µmol/L. Few additional comparative data were available between studies. Bias analysis indicated an acceptable level of agreement (within two SDs or 95% acceptability limits) between these devices’ performance and HPLC.

Of note, we identified an additional report examining “vitamin A… in plasma” as measured by the CRAFTi compared with HPLC (Craft, 2005). The correlation between methods was 0.82. However, these data came from a summary of a poster submitted to a conference, and we were unable to find the full version of the poster; therefore, the report was excluded from our primary results and is not in Table 5a above.

Four studies analyzed either human or cow’s milk samples with the iCheck Fluoro (Table 5b). The device performance varied: some studies reported lower, equivalent, or higher retinol values than those of HPLC. The R^2^ values for the correlation between the device and HPLC ranged between 0.35 and 0.79 after adjustment for milk fat content.

In one study (Schweigert et al., 2011b), the authors tested increasingly diluted cow’s milk samples with 3.5% fat by using the iCheck, which showed linearity at an R^2^ of > 0.99 between 100 and 2500 µg RE/L. The same study also showed a positive correlation between percentage milk fat and µg RE/L milk with the iCheck Fluoro. Precision was tested over an operational range of 60 to 600 µg RE/L, and the inter-assay CV was < 3.5% (not shown in table).

RBP was measured in blood with two field-friendly immunoassays reported across three studies comparing a portable device to a reference method (Table 5c). A rapid enzyme immunoassay (RBP-REI), available from Scimedx Corp, was able to detect serum RBP within a range of 10–40 µg/mL, which correlated with the HPLC results (R^2^ = 0.79 to 0.86) (Hix et al., 2004, Hix et al., 2006). The RBP-REI assay was also compared with another portable, laboratory-based device (index 2), a commercially available radial immunodiffusion plate reader (RID; The Binding Site, San Diego), by measuring RBP in 40 serum samples (Hix et al., 2004). Compared with the higher R^2^ values in validation against HPLC (R^2^ = 0.82 and 0.86; Table 5c), the RBP-REI had a lower, but still acceptable, correlation with the RID method (R^2^ = 0.73; linearity: y = 0.50x + 0.45) (not shown in table). Comparison of RID and HPLC indicated a slightly lower correlation (R^2^ = 0.71) (Table 5c). Other validity and precision data were not reported for the comparisons of REI vs. RID, or RID vs. HPLC. From the current manufacturer’s website (accessed date: March 15, 2021) (The Binding Site, 2020), RBP was not listed among the human proteins for assessment with the RID plate reader.

A semi-quantitative antigen–antibody binding assay allowed for detection of low concentrations of RBP in serum samples (Ciaiolo et al., 2015). However, because only six samples were used for validation, drawing a conclusion regarding the efficacy of this method is difficult.

We identified two microfluidics-based devices, the EE-µPAD (Lee et al., 2016) and the Tidbit with HYPER filtration (Lu and Erickson, 2017, Lu et al., 2017, Lu et al., 2018), both of which were able to separate whole blood into serum, detect VAD at high sensitivity and specificity with respect to the reference ELISA test, and send results to a mobile device (Table 5d). We note that the ELISA test may not be a suitable reference method for assessing VAD, owing to inherent problems with antibodies to RBP. Neither device is currently on the market.

Although we identified several portable dark adaptometers (Table 1b), we found only one validation study between a portable dark adaptometer, the Scotopic Sensitivity-Tester 1 by LKC Technologies, and a reference standard, the Goldmann-Weekers dark adaptometer used in clinical settings (Peters et al., 2000) (Table 5e). The portable device was comparable to the reference standard in its sensitivity in identifying elevated final thresholds for dark adaptation, with a correlation (R^2^) of 0.77. However, this study was performed in the US in an eye clinic, and it remains to be tested and compared with the reference standard in field settings.

The iCheck Fluoro, a portable fluorometer, was used to measure bovine blood samples for retinol (Table 5f). Compared with HPLC, mean differences in whole blood, plasma, or serum retinol ranged from −0.01 µmol/L to 26.5 µmol/L, and the iCheck generally displayed higher values than HPLC. The correlation between the iCheck Fluoro and HPLC was positive, ranging in R^2^ values from 0.61 to 0.96. Weaker correlations were observed in cows (range: 0.78–88) than calves (0.90–0.96).

Raila and et al. (2017) also compared the correlation between bovine whole blood retinol (n = 10) and plasma retinol (n = 10), both measured by the index test iCheck Fluoro, and found a significant positive correlation (R^2^ = 0.87) (Raila et al., 2017). No studies reported sensitivity and specificity, or distinguished specific %CVs. Bias analysis indicated acceptable agreement between the device performance and HPLC.

The iCheck Carotene, a portable photometer, was used to measure carotenoids in bovine whole blood and plasma. Mean differences in beta-carotene concentration ranged from −0.29 mg/L to 0.26 mg/L in plasma samples in cows and calves (Table 5g). The correlation between iCheck Carotene and HPLC was high, with R^2^ between 0.93 and 0.99. No studies reported sensitivity, specificity, or specific %CVs. Bias analysis revealed an acceptable level of agreement between the device performance and that of HPLC.

## Future perspectives and recommendations

### Gaps and recommendations

On the basis of our review of the literature, portable devices fell into five categories:

1. Portable fluorometers
2. Portable photometers
3. Field-friendly immunoassays and/or microfluidics-based devices
4. Slit lamps
5. Dark adaptometers

We found that, although many portable devices for quantifying vitamin A have been developed and described, only a few devices appear to be currently on the market or commercially available; of these, only two had easily accessible performance criteria information on the manufacturers’ websites related to vitamin A measurement. Studies tended not to report on portable device characteristics.

Some major gaps involve the lack of data reported by studies. Few studies have reported the portable device’s sensitivity and specificity in detecting VAD compared with the reference standard method—a necessary metric for validation and adoption by randomized trials. Furthermore, only the iCheck devices were assessed in more than two studies; other devices should be analyzed further for validation.

### Minimal set of criteria for point-of-need devices

See Fig. 2.

**Fig. 2:**
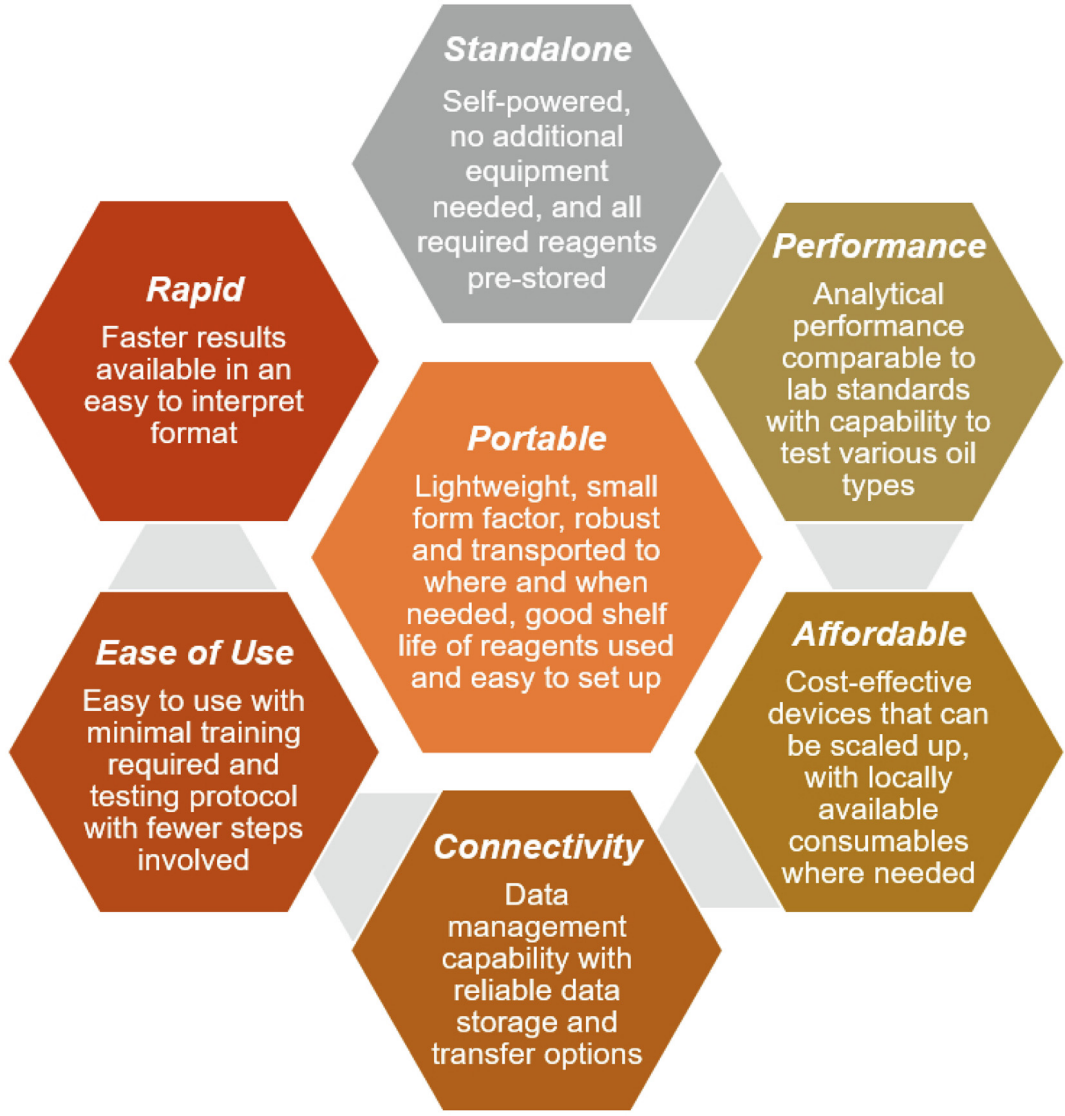
Minimal set of criteria for point-of-need devices. Adapted with permission (Huey, 2022).

The device should:

1. Be lightweight with a small form factor for easy transport to the necessary location as needed.
2. Be standalone without needing additional equipment and self-powered, and should pre-store all the required reagents for the test, and use common reagents that are available on the market.
3. Be easy to use with minimal processing steps in the protocol, and should require minimal training effort.
4. Have analytical performance (e.g., %CV < 5% or within Bland Altman 95% limits of agreement) comparable to those of the current laboratory standards, with a capability to test various biological samples.
5. Be affordable and capable of scaling up with locally available consumables where needed.
6. Be able to connect to the internet or an external hard drive with a built-in data management system to allow the test results to be reliably stored and transferred.
7. Be able to output test results quickly and present in a format that is easy to interpret.

## Conclusions

In this review, we identified 25 portable methods or devices for a variety of biological sample types including those of human (blood, milk, and eye/vision) and animal (blood and milk) origin. These included nine methods measuring biochemical markers of vitamin A or VAD (serum retinol, RBP, milk retinol, retinyl palmitate, and retinyl esters) and 17 portable methods measuring functional biomarkers (measures of eye health, for example dark adaptation).

The iCheck devices, including iCheck Carotene and iCheck Fluoro—for measuring total carotenoids or beta-carotene, or for measuring retinol, retinyl palmitate, retinyl acetate, or other esters, respectively, in blood or milk—were the only devices with manufacturer-reported performance metrics as well as the most information and data available to ascertain the method’s accuracy and precision with respect to those of a gold standard such as HPLC. These methods, in addition to the CRAFTi portable fluorometer, as compared with HPLC, were thus considered acceptable for measuring both blood and milk for biochemical biomarkers of vitamin A and detecting vitamin A deficiency.

In measuring human or cow milk samples’ retinol concentration, the iCheck Fluoro had variable performance across studies, including both lower and higher values than the gold standard HPLC, thus leading to weaker correlation values than those calculated for blood samples. However, the mean differences were<1 µmol/L, and the values were considered to be within the expected variance. Correlation was improved by diluting the samples; dilution may be required for higher accuracy when the portable method is used.

Several portable immunoassays (RBP-REI, RID, general immunoassay) and microfluidics-based methods (EE-µpad, TIDBIT with or without HYPER platform) for measuring RBP in human blood had acceptable correlations with HPLC reference methods and similar detection of VAD. However, these assays appeared not to be commercially available.

One study has measured eye function with a portable dark adaptometer (Scotopic Sensitivity Tester-1), which had comparable results to the gold standard, a Goldmann-Weekers dark adaptometer. However, field studies using this device in comparison to a reference remain to be performed. Given the importance of eye health as a functional indicator of vitamin A deficiency, this gap in the literature is substantial.

Finally, the iCheck Fluoro was used for measuring bovine blood samples for retinol. Generally, the retinol values were higher than those in samples tested by HPLC. Retinol measurements in calves appeared to have stronger correlations than retinol in cow’s blood.

Several studies examined the accuracy of the iCheck Carotene, as compared with HPLC, in determining carotenoid content in cow’s blood. Strong correlations with acceptable levels of agreement were observed between device performance and HPLC performance.

In summary, the iCheck devices are commercially available and are acceptable for measuring vitamin A in blood and milk, on the basis of the available data. Many of the other identified devices were proofs of concept and not yet commercially available. Several gaps remain, including studies comparing the other portable devices against a gold standard, particularly for functional indicators of vitamin A status/deficiency; available manufacturer-reported device performance criteria against which to compare the results of future investigations; and more comprehensive reporting of sensitivity, specificity, precision, and other validation metrics.

## CRediT authorship contribution statement

**Samantha L. Huey:** Data curation, Formal analysis, Investigation, Methodology, Writing – original draft, Writing – review & editing. **Jesse T. Krisher:** Data curation, Formal analysis, Investigation, Methodology, Writing – original draft, Writing – review & editing. **David Morgan:** Project administration, Supervision, Writing – review & editing. **Penjani Mkambula:** Project administration, Writing – review & editing. **Bryan M. Gannon:** Conceptualization, Methodology, Writing – review & editing. **Mduduzi N.N. Mbuya:** Writing – review & editing. **Saurabh Mehta:** Conceptualization, Funding acquisition, Methodology, Supervision, Writing – review & editing.

## Declaration of Competing Interest

The authors declare that they have no known competing financial interests or personal relationships that could have appeared to influence the work reported in this paper.
