# Supplementary material for: A review of portable quantitative and semi-quantitative devices for measurement of vitamin A in biological samples
Source: Curr Res Biotechnol. 2022;4:253–74. doi: 10.1016/j.crbiot.2022.04.003 (PMC9407042; doi:10.1016/j.crbiot.2022.04.003)
Supplement: Supplementary data 1 [file mmc1.docx]

| **Supplementary Table 1. MEDLINE (PubMed) search strategy** | | |
| --- | --- | --- |
| **#** | **Search string** | **Records:**  **December 16, 2020** |
| **1** | Vitamin a deficiency[mesh] OR carotenoids[mesh] | 88,102 |
| **2** | Vitamin a deficien*[tiab] OR vitamin a insufficien*[tiab] OR vitamin a replet*[tiab] OR vitamin a sufficien*[tiab] OR retinol[tiab] OR hypovitaminosis a[tiab] OR avitaminosis a[tiab] OR serum vitamin a[tiab] OR serum retinol[tiab] OR retinol-binding protein[tiab] OR RBP[tiab] OR retinol activity equivalent*[tiab] OR retinal[tiab] OR carotenemia[tiab] | 178,591 |
| **3** | #1 OR #2 | 251,987 |
| **4** | Point of care[mesh] OR point-of-care systems[mesh] | 15,303 |
| **5** | Points of care[tiab] mobile[tiab] OR portable[tiab] OR device[tiab] OR spectroscopy[tiab] OR paper[tiab] OR point of care[tiab] OR fluorometry[tiab] | 1,416,125 |
| **6** | #4 OR #5 | 1,421,765 |
| **7** | Blood[mesh] OR dried blood spot testing[mesh] OR saliva[mesh] OR tears[mesh] OR liver[mesh] OR skin[mesh] | 1,787,902 |
| **8** | Blood[tiab] OR saliva*[tiab] OR tears[tiab] OR tear[tiab] OR liver[tiab] OR skin[tiab] OR dark adapt*[tiab] OR ocular[tiab] OR pupillary[tiab] OR ophthalm*[tiab] OR breast milk[tiab] OR breastmilk[tiab] OR human milk[tiab] | 3,535,356 |
| **9** | #7 OR #8 | 4,497,984 |
| **10** | #3 AND #6 AND #9 | 2,683 |
| **11** | Acne vulgaris[mesh] OR Dermatologic Agents/therapeutic use*[mesh] OR retinal detachment*[mesh] OR isotretinoin[mesh] | 127,898 |
| **12** | Acne[tiab] OR tretinoin[tiab] | 17,522 |
| **13** | #11 OR #12 | 135,102 |
| **14** | #10 NOT #13 | 2,502 |
|  |  |  |
